# Ex-Vivo Trans-Corneal and Trans-Scleral Diffusion Studies with Ocular Formulations of Glutathione as an Antioxidant Treatment for Ocular Diseases

**DOI:** 10.3390/pharmaceutics12090861

**Published:** 2020-09-10

**Authors:** María Sebastián-Morelló, Adrián M. Alambiaga-Caravaca, María Aracely Calatayud-Pascual, Vicent Rodilla, Cristina Balaguer-Fernández, María Miranda, Alicia López-Castellano

**Affiliations:** Facultad de Ciencias de la Salud, Instituto de Ciencias Biomédicas, Universidad Cardenal Herrera-CEU, CEU Universities, C/Santiago Ramón y Cajal, s/n., Alfara del Patriarca, 46115 Valencia, Spain; maria.sebastian@uchceu.es (M.S.-M.); adrian.alambiagacaravaca@uchceu.es (A.M.A.-C.); maria.calatayud@uchceu.es (M.A.C.-P.); cbalaguer@uchceu.es (C.B.-F.); mmiranda@uchceu.es (M.M.)

**Keywords:** glutathione, antioxidants, ocular drug delivery, hen’s egg-choriollantoic membrane test (HET-CAM), ocular diffusion studies

## Abstract

Exposure to sunlight and contact with atmospheric oxygen makes the eye particularly susceptible to oxidative stress, which can potentially produce cellular damage. In physiological conditions, there are several antioxidant defense mechanisms within the eye. Glutathione (GSH) is the most important antioxidant in the eye; GSH deficit has been linked to several ocular pathologies. The aim of this study was to explore the potential for newly developed formulations allowing controlled delivery of antioxidants such as GSH and vitamin C (Vit C) directly to the eye. We have investigated the stability of antioxidants in aqueous solution and assessed ex-vivo the diffusion of GSH through two ocular membranes, namely cornea and sclera, either in solution or included in a semisolid insert. We have also carried out the hen’s egg-chlorioallantoic membrane test (HET-CAM) to evaluate the ocular irritancy of the different antioxidant solutions. Our results showed that GSH is stable for up to 30 days at 4 °C in darkness and it is not an irritant to the eye. The diffusion studies revealed that the manufactured formulation, a semisolid insert containing GSH, could deliver this tripeptide directly to the eye in a sustained manner.

## 1. Introduction

Oxidative stress was defined by Helmut Sies as “a change in the prooxidant-antioxidant balance in favor of the former” [1]. Oxidative stress stimulates the production of highly reactive oxygen species (ROS), potentially leading to cellular damage. Our eyes are especially susceptible to oxidative stress because they are greatly exposed to sunlight and atmospheric oxygen [2]. Indeed, alterations in the cellular redox state within the eye are believed to contribute to the pathogenesis of many ocular diseases [3].

Under normal physiological conditions, ocular tissues have several intrinsic antioxidants to overcome the oxidative stress generated as a result of normal cell metabolism. The two key antioxidants in the eye are glutathione (GSH) and vitamin C (Vit C) [4]. While ascorbic acid (Vit C) is found in the corneal epithelium, GSH is found mainly in the lens [5,6].

Vit C is one of the most important antioxidants in the cornea [7] and as it is neither synthesized nor stored in the human body, dietary intake is essential to maintain its adequate levels. Vit C is also found in high concentrations in the aqueous humor [4]. Dietary intake of antioxidants like Vit C has been associated with protective effects against the incidence of three major eye diseases linked to oxidative stress: cataracts, age-related macular degeneration, and glaucoma [8].

GSH is a tripeptide of L-glutamate, L-cysteine, and glycine [9], and is considered the most important intracellular endogenous antioxidant in living organisms and the first line of defense against oxidative stress. GSH exists in the form of reduced thiol (GSH) and oxidized disulfide (GSSG) [10]. GSH is formed in a two-step enzymatic process including, first, the formation of γ-glutamylcysteine from glutamate and cysteine (Cys), a reaction catalyzed by the enzyme γ-glutamylcysteine synthetase; and second, GSH synthetase (GS) intervenes to catalyze the condensation of γ-glutamylcysteine and glycine to generate GSH [11].

GSH levels in the eyes are maintained by a combination of GSH dietary uptake, de novo synthesis from its precursor amino acids, GSH regeneration from GSSG, and GSH transport [4]. The transport of GSH through membranes is regulated by transporters and occurs according to a concentration gradient; however, the regulatory mechanisms of those GSH transporters are still not well understood [12]. 

Deficit in GSH has been linked to retinal pathologies. The retina has a high need for antioxidant protection because it is exposed to high oxygen pressure (oxygen consumption in the retina is the highest among all human tissues) as well as UV and blue light. As photoreceptor membranes are rich in polyunsaturated fatty acids which are readily oxidized and there is a complex choroidal blood flow autoregulation, antioxidant protection is of utmost importance [13]. In the retina, Müller cells produce GSH but with age, production of GSH is reduced. This is related to mitochondrial damage, reduced cell viability, and progression of several retinopathies in the elderly, such as diabetic retinopathy, glaucoma, or age-related macular degeneration [14]. Alterations in GSH metabolism have also been linked to hereditary retinal degenerations such as retinitis pigmentosa [15]. GSH is also directly associated with cataract development. It is known that with increasing age, GSH in the lens nucleus decreases and the lens may become more susceptible to cataract formation [16].

As GSH has poor bioavailability, interventions aimed to increase GSH concentrations in different tissues have relied on administration of GSH, monoesters, or alternative precursors [17]. 

It has been known for years that GSH import in mammals involved its extracellular degradation, uptake of its constituents, and re-synthesis inside the cells. However, the existence of specific transporters for GSH has been demonstrated in different tissues [18]. Several studies have reported the beneficial effect of direct oral or nasal GSH administration in inflammation, alterations of the immune system, or Parkinson’s disease [19,20,21]. In addition, it has been shown that oral administration of antioxidant mixtures containing GSH were able to reduce photoreceptor cell death in an animal model of retinitis pigmentosa [22,23]. All this evidence supports the possible beneficial effects of GSH administered directly to ocular tissues.

The most common route for drug administration to the eyes is topical administration, as application is easy, patient compliance is high, and dosage and secondary effects are minimized [24]. However, due to the eye’s inherent anatomical and physiological barriers, diffusion of drugs topically applied to the eye is very poor [25]. Typically, drug bioavailability is estimated to be less than 5% of the drug administrated by drop instillation [25]. 

Ocular inserts are solid or semisolid devices that can deliver drugs to the eye [26,27]. These inserts provide accurate dosing, reduced systemic diffusion, and in some cases, better patient compliance due to reduced administration frequency and lower incidence of visual and systemic side effects [28,29]. Furthermore, ophthalmic inserts may facilitate drug diffusion and efficacy as permanence of the drug in the surface of the eye becomes increased.

The aim of this study was to evaluate the potential for developing formulations allowing controlled delivery of antioxidants such as GSH and Vit C directly to the eye. The study assessed the stability of antioxidants (GSH and Vit C) in aqueous solution and evaluated their ocular irritancy. Furthermore, the potential of ocular antioxidant formulations was assessed by diffusion studies of GSH through cornea and sclera, either from solution or from a purposely manufactured semisolid insert.

## 2. Materials and Methods

### 2.1. Reagents

Most chemicals (GSH, Vit C, iodoacetic acid, potassium hydroxide (KOH), potassium bicarbonate (KHCO_3_) 1-fluoro-2,4-dinitrobencene (DNFB), disodium hydrogen phosphate, sodium dihydrogen phosphate, sodium chloride, and bidestilled water) were purchased from Sigma-Aldrich (Madrid, Spain). Acros Organics (Morris Plains, NJ, USA) supplied m-cresol, and perchloric acid (PCA) was obtained from Panreac (Barcelona, Spain).

Phosphate buffered solution (PBS) was prepared in the laboratory and its pH adjusted to 7.5 ± 0.1 with hydrochloric acid (HCl) 5 N or sodium hydroxide (NaOH) 5 N.

Most reagents for high performance liquid chromatography (HPLC) (methanol, water, and glacial acetic acid) were purchased from J.T. Baker^®^ (Deventer, Netherlands), but sodium acetate was purchased from Sigma-Aldrich (Madrid, Spain).

Reagents for insert preparation (hydroxypropyl methylcellulose 4500 (HPMC), polyvinylpyrrolidone K30 (PVP-K30), polyethylene glycol (PEG) and glycerol) were supplied by Guinama (Valencia, Spain).

### 2.2. Ocular Tolerance Test (HET-CAM Test) Fertile

Fertile eggs from White Leghorn hens were supplied by Granja Santa Isabel (Cordoba, Spain) and incubated at 37 °C and 60% relative humidity. Eggs were incubated in an incubator (Covatutto 24 digitale, Novital S.r.l, Lonate Pozzolo, Italy) coupled to an egg turner (Girauova automatic, Novital S.r.l, Lonate Pozzolo, Italy) which automatically rotated the eggs to ensure correct embryo development. After being incubated for 8 days, a circular cut was made on the eggshell using a rotatory saw. After moistening the inner membrane with a 0.9% NaCl solution, this was carefully removed to expose the chorioallantoic membrane (CAM). Aliquots (300 µL) of GSH (5 mg/mL and 10 mg/mL, and 5 mg/mL GSH with 3 mg/mL Vit C) were placed on the CAM of different eggs. Negative and positive controls were solutions of 0.9% NaCl and 0.1 N NaOH, respectively. Irritation score (IS) was calculated (1) by monitoring and recording the time of appearance of each of the following endpoints: hemorrhage (tH, bleeding from the vessels), vascular lysis (tL, blood vessel disintegration), or coagulation (tC, intravascular and extravascular protein denaturation) of CAM vessels over a period of 300 s [30].

(1) $$\mathrm{IS}\;=\;\frac{\left(301\;-\;\mathrm{tH}\right)\times 5}{300}\;+\;\frac{\left(301\;-\;\mathrm{tL}\right)\times 7}{300}\;+\;\frac{\left(301\;-\;\mathrm{tC}\right)\times 9}{300}$$

The values of damage were classified by means of IS as IS < 1 (non-irritant), 1 ≤ IS < 5 (mild irritant), 5 ≤ IS < 10 (moderately irritant), or IS > 10 (severe irritant).

### 2.3. Stability Studies of GSH

A GSH solution in PBS (5 mg/mL) adjusted at pH 6.5 was prepared to evaluate GSH stability in solution under different conditions. The role of Vit C as an antioxidant was also assessed by preparing an additional GSH solution (5 mg/mL) containing 3 mg/mL of Vit C [22].

The effects of light and temperature on the stability of GSH solutions were addressed by storing them at 4 °C and 25 °C in darkness or under illumination and sampling and analyzing them at 0, 2, 7, 14, and 30 days.

### 2.4. Ocular Insert with GSH

A semisolid ocular insert using bioadhesive polymers (HPMC, PVP, and PEG) [31] with glycerol as a plasticizer and GSH in the matrix was prepared using a solvent casting method. The polymers were weighed and dissolved by gently stirring in the necessary amount of water (quantum satis, qs) (Table 1) to make a final volume of 10 mL. The corresponding amount of glycerol was then added to the solution which was continuously stirred at room temperature for 24 h [32,33] before adding GSH.

### 2.5. Ex-Vivo Ocular Diffusion Studies

Whole eyes were obtained after sacrifice from two-month-old hybrid albino rabbits of either sex [34,35,36]. The eyes were immediately rinsed in saline solution and any muscle remaining attached was cut away with scissors. An incision along the sclera-limbo junction was made to obtain the cornea and sclera from each eye, which were then used as membranes for diffusion studies [36,37]. The experimental protocol was approved by the Ethical Committee of University CEU Cardenal Herrera (Ref. 2011/010) and by the Conselleria d’Agricultura, Pesca I Alimentació, Generalitat Valenciana (Ref. No. 2017/VSC/PEA/00192). Prior to sacrifice, animals were housed, fed, and handled according to current animal welfare principles (Spanish Royal Decree 1201/2005, (BOE 2005)).

GSH diffusion through cornea and sclera was assessed using vertical standard diffusion cells (Franz type) with a diffusion area of 0.567 ± 0.008 cm^2^ (DISA, Milan, Italy) [38]. The membrane (i.e., cornea or sclera) was placed in the diffusion cell with the external part facing the donor compartment. The receptor compartment of the diffusion cells (in contact with the internal side of the cornea or sclera) was filled to its capacity (4.2 ± 0.1 mL) with PBS (pH 7.4) at 37.0 ± 0.1 °C. To prevent possible boundary layer effects, PBS was stirred with a rotating Teflon-coated magnet. The donor compartment was filled with 0.5 mL of GSH solution (5 mg/mL or 10 mg/mL) or 0.5 mL of the previously manufactured semisolid insert of GSH (10 mg/mL).

Samples (180 μL) were taken from the receptor compartment every 15 min during the first hour, and then every 30 min during the following 2 h [39]. Immediately after the sample was withdrawn, the volume was replaced with 180 μL of PBS to ensure that sink conditions in the receptor compartment were maintained [34,35,40]. To ensure preservation of the sample until analysis, a 20 μL volume of PCA (20% solution) was added to each sample.

### 2.6. Determination of GSH

GSH concentration in each experimental sample was determined according to published procedures [30]. Briefly, HPLC analysis was carried out using a Gilson system (Gilson 322 pump, Gilson 150 UV/VIS detector, Gilson 864 degasser, and Gilson 234 automatic injector) (Gilson, Middleton, WI, USA). Data were acquired and processed on a Unipoint™ software system. A Kromasil^®^ Amino 5 µm, 250 × 4.6 mm column was purchased from Análisis vínicos (Barcelona, España). All solutions were filtered using cellulose membrane filters (Sartorius Stedim Biotech (Madrid, España) with a 0.2 µm pore diameter.

Mobile phase A was prepared with 80:20 methanol:water (*v/v*) and mobile phase B was prepared with a 20% solution of sodium acetate trihydrate 4 M and 756 mL/L glacial acetic acid in water and 80% of phase A. Initial chromatography conditions (1.0 mL/min, 80% mobile phase A and 20% mobile phase B) were maintained for 10 min which were followed by a linear gradient to 95% mobile phase B for 40 min. Initial chromatography conditions were then pumped through the column for 10 min to prepare the system for the next sample. The wavelength used was 365 nm. The range of the UV detector used was 0.005 and the response time was 5 s.

The stock solution for calibration (10 mM GSH in PBS) was prepared daily and stored in a refrigerator at 4 °C until required. Standards for calibration curves were prepared daily using the following GSH concentrations: 0 µM, 20 µM, 40 µM, 60 µM, 80 µM, and 100 µM. To a 180 µL volume of each of these standards, 20 µL of a 20% solution of perchloric acid was added to acidify the solution. Both samples and standards were prepared in 1.5 mL Eppendorf tubes and were derivatized prior to analysis, following the protocol described by Reed et al. [41].

### 2.7. Data Analysis

Data were always expressed as mean ± SD. Data were assumed to be normally distributed and homoscedasticity was assessed with Levene’s test. Homoscedastic data were statistically analyzed by means of a one-way ANOVA and post-hoc multiple comparison tests (Scheffeé). When unequal variances were present, data were analyzed using the Brown—Forsythe ANOVA and multiple comparisons were carried out using the Dunnett T3 test. For two group comparisons the Student *t*-test (homoscedastic data) or the Welch test (heteroscedasticity) were used. The level of significance was fixed at *p* < 0.05.

## 3. Results and Discussions

The hen’s egg test on the chorioallantoic membrane (HET-CAM) (an alternative to the in vivo Draize rabbit eye test) was carried out to assess the potential irritancy of GSH and Vit C solutions.

**Figure 1:**
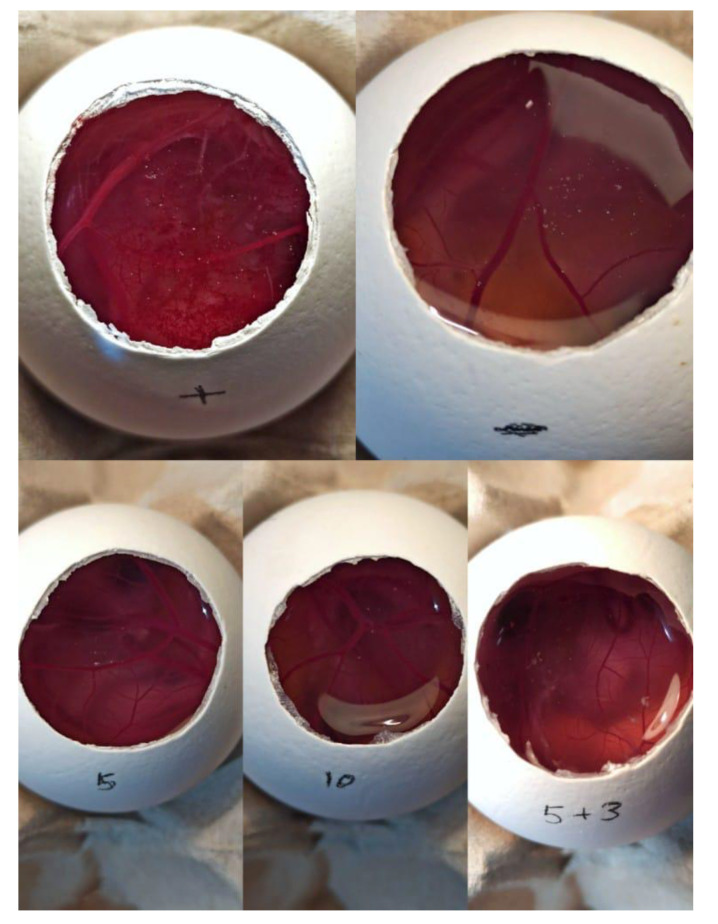
Photograph of the hen’s egg-choriollantoic membrane test (HET-CAM) test results to evaluate the irritancy of glutathione (GSH) solutions. In clockwise direction, positive (0.1 N NaOH) and negative (0.9% NaCl) controls, 5 mg/mL and 10 mg/mL GSH solutions, and 5 mg/mL GSH containing 3 mg/mL Vit C.

The HET-CAM test revealed that GSH solutions (10 mg/mL, 5 mg/mL) and the solution of 5 mg/mL GSH with 3 mg/mL of Vit C, did not produce hemorrhages, lysis, or coagulation (Figure 1). The IS for the negative control (0.9% NaCl) and for all formulations assayed was zero. Therefore, all antioxidant solutions were considered to be non-irritant. In contrast, the IS for the positive control (NaOH 0.1 N) was 45 s. These results are consistent with similar studies which evaluated the irritancy of antibiotics or hormones using the HET-CAM test [42,43].

Stability studies were carried out using two solutions: 5 mg/mL GSH and 5 mg/mL GSH containing 3 mg/mL Vit C. Different preservation conditions were also studied (25 °C with light exposure, 25 °C in darkness and 4 °C in darkness). The results of stability experiments revealed that there were differences across times in the samples stored at room temperature for GSH and GSH with Vit C (in all cases *p* < 0.001). Statistically significant lower concentrations of GSH were seen from day 7 onwards when samples were stored at room temperature. However, samples remained stable in both cases (GSH and GSH with Vit C (*p* = 0.176)) for up to 30 days when stored at 4 °C in darkness (*p* = 0.445) (Table 2).

We also compared differences in stability between GSH and GSH with Vit C at different storage times for each condition. Although GSH stability decreases with time in the three storage conditions, this is more pronounced in samples stored at room temperature, either with or without light. In these cases, statistical differences are particularly seen from day 14 onwards (*p* < 0.05). However, when GSH and GSH + Vit C samples were stored at 4 °C in the dark, no statistical differences were found (*p* > 0.05) (Table 2).

In summary, these data showed that better stability was achieved when samples were stored at 4 °C in darkness, as no statistically significant differences were detected across storage time. Significantly lower levels of GSH were measured when samples were stored at room temperature. Although some studies have shown that antioxidants like vitamins C and E can compensate for low levels of GSH in the lens, thus protecting against oxidative insults [44], it seems that in our studies, Vit C is readily oxidized at room temperature [45,46] and as GSH intervened non-enzymatically to reduce Vit C, lower levels of GSH were detected in these cases. The role of GSH in reducing oxidized Vit C was demonstrated as far back as 1976 by Foyer and Halliwell who showed that in absence of dehydroascorbate reductase, GSH was responsible for the reduction of dehydroascorbate back to ascorbate (Vit C) [47]. Although these data referred to chloroplasts, similar biochemical reactions have been described in other living organisms [48] and one can expect that they can also take place in the test tube, as in our case.

Following these experiments, ex-vivo diffusion studies of GSH solutions through cornea and sclera were performed. All our experiments have been carried out in freshly-obtained tissue because although sometimes diffusion studies have been carried out using frozen-thawed tissue [49,50] it has been shown that frozen tissue suffers significant histological changes which affect diffusion [36]) and that particularly with small substances (GSH is a relatively small tripeptide) higher diffusion rates across frozen-thawed tissue are observed when compared to fresh tissue [50].

**Figure 2:**
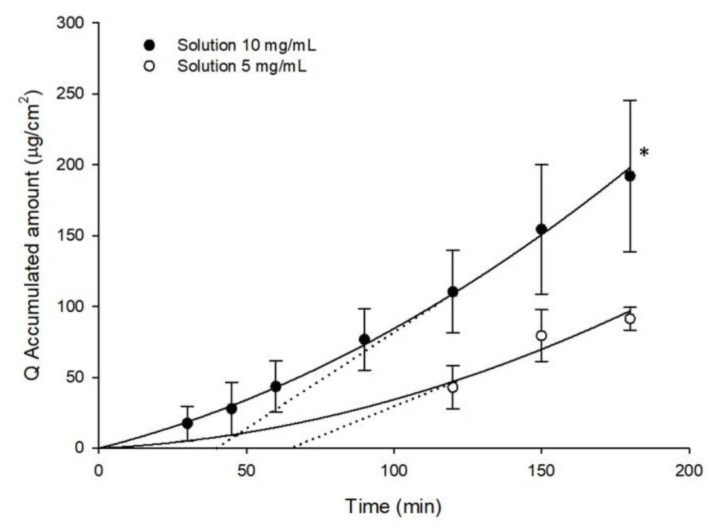
Accumulated amounts of GSH in the receptor compartment (Q, μg/cm^2^) as a function of time, obtained from samples employed in ex-vivo trans-corneal diffusion studies of different solutions. The dotted line represents the estimated lag time (t_0_) calculated by intersecting with the *x*-axis of the linear regression. (Mean ± SD; *n* ≥ 3) * *p* < 0.05.

**Figure 3:**
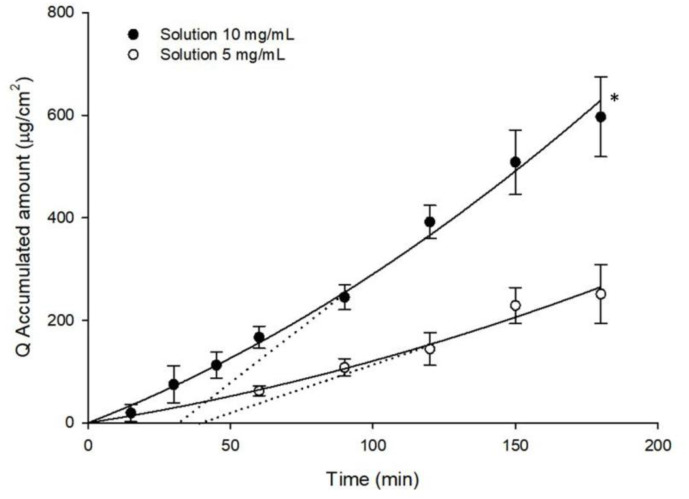
Accumulated amounts of GSH in the receptor compartment (Q, μg/cm^2^) as a function of time, obtained from samples employed in ex-vivo trans-scleral diffusion studies of different solutions. The dotted line represents the estimated lag time (t_0_) calculated by intersecting with the *x*-axis of the linear regression. (Mean ± SD; *n* ≥ 3) * *p* < 0.05.

There were statistically significantly differences between the accumulated amount of GSH in the receptor compartment obtained from the 10 mg/mL and 5 mg/mL solutions in ex-vivo trans-corneal diffusion studies (191.89 ± 53.44 µg/cm^2^ and 91.29 ± 7.97 µg/cm^2^, respectively; *p* < 0.05) (Figure 2). The estimated lag time (t_0_) was calculated by intersecting the linear regression curve obtained with the *x*-axis (Figure 2 and Figure 3).The estimated lag period of GSH permeation for the 5 mg/mL solution was 66 min whereas it was 40.6 min for the 10 mg/mL solution. These differences are related to the poor detection of GSH with the 5 mg/mL GSH solution. As we can see in Figure 2, the first time-point at which accumulated GSH could be measured corresponds to 120 min and hence, extrapolation of the regression curve to intersect the *x*-axis lacks required precision (Figure 2).

The accumulated amounts of GSH in the receptor compartment obtained in ex-vivo trans-scleral diffusion studies carried out with both solutions are shown in Figure 3. Accumulated amounts with 10 mg/mL solution of GSH were significantly higher than those obtained with the 5 mg/mL solution of GSH (596.74 ± 78.05 μg/cm^2^ and 251.29 ± 57.69 μg/cm^2^, respectively; *p* < 0.05). For sclera, the estimated lag time for GSH diffusion was 39 min for the 5 mg/mL solution and 32.2 min for the 10 mg/mL solution. As before, the absence of data (GSH could not be quantified in the earliest part of the curve) may account for the small differences seen in lag-time (Figure·3) As the amount of accumulated GSH in the receptor compartment was significantly higher when the 10 mg/mL GSH solution was used, both in cornea and sclera, the ocular insert was prepared and formulated with a GSH concentration of 10 mg/mL.

**Figure 4:**
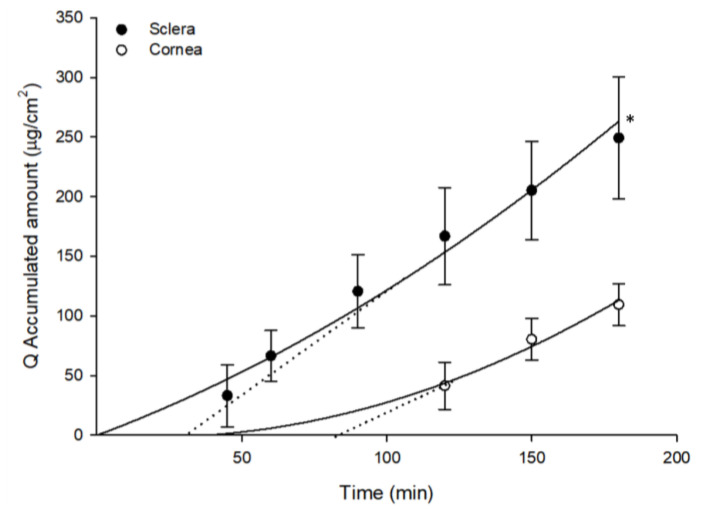
Accumulated amounts of GSH in the receptor compartment (Q, μg/cm^2^) as a function of time, obtained from samples employed in ex-vivo trans-corneal and trans-scleral diffusion studies with the formulated insert. The dotted line represents the estimated lag time (t_0_) calculated by intersecting with the *x*-axis of the linear regression. (Mean ± SD; *n* ≥ 3) * *p* < 0.05.

A semisolid insert of GSH was formulated in order to develop a controlled delivery system for GSH. Our results show that GSH diffused more efficiently through sclera than through cornea (*p* < 0.05) (Figure 4). The total accumulated amount in the receptor compartment when cornea was used as a membrane was 109.46 ± 17.61 μg/cm^2^, whereas for sclera the total accumulated GSH was 249.09 ± 51.17 μg/cm^2^. The estimated lag period for diffusion of GSH when we used the insert was 82.5 min in cornea and 31.4 min in sclera.

**Figure 5:**
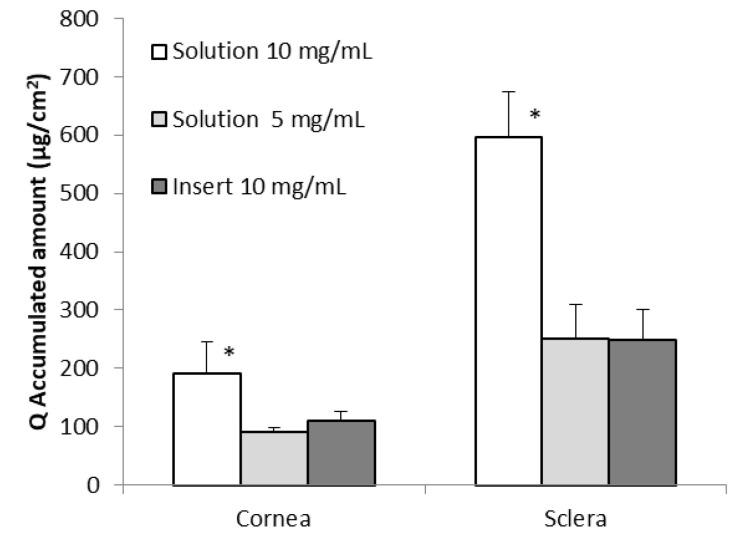
Accumulated amount of GSH in the receptor compartment after 3 h of experiment (Q, μg/cm^2^) obtained from samples of ex-vivo trans-corneal and trans-scleral diffusion studies performed with different formulations. (Mean ± SD; *n* ≥ 3) * *p* < 0.05.

The amount of GSH permeating through sclera was greater than that permeating through cornea in the ex-vivo diffusion studies performed with all the formulations assayed (*p* < 0.05) (Figure 5). This could be attributed to the different characteristics of the two tissues. The cornea is a complex tissue with five different layers [51]. The outer layer is a stratified squamous non-keratinized epithelium consisting of no less than five layers of cells with varying affinity to hydrophilic and lipophilic molecules [52]. These characteristics, particularly the presence of the corneal epithelium, make diffusion of any hydrophilic molecule across the cornea rather difficult [53]. Histologically, the sclera is composed mainly of collagen fibers [54] forming a dense connective tissue and thus GSH, which is a rather hydrophilic molecule, can permeate through sclera much more easily than it does through cornea.

If we focus on the anatomy of the eye, the sclera is only separated from the retina by choroid [23]. Our results suggest that these GSH formulations may be used to target the retinal tissue if we assume that GSH is able to reach the retina despite the choroid being a highly vascularized tissue. In fact, several studies have used different liposomal formulations with pegylated glutathione in order to target retina and other neuronal tissues [55,56,57].

The highest accumulated GSH values in the receptor compartment were measured when sclera was used as a membrane and a 10 mg/mL GSH solution was used (Figure 5). Similar accumulated GSH values were measured when a 5 mg/mL GSH solution or 10 mg/mL GSH in a semisolid insert were used (Figure 5). No statistical differences were found between the values measured in this case (*p* < 0.05).

In order to determine the permeability of GSH through cornea and sclera we calculated the permeability coefficients (Kp) of GSH under the different assay conditions used in ocular diffusion studies (Table 3). Kp values (Table 3) were calculated by means of the following equation.

Kp = J/C(2)

where Kp is the permeability coefficient of the drug (cm/h), J is flux of GSH though the membrane, obtained from the slope between the accumulated amount of the drug in the receptor compartment (Q, mg/cm^2^) versus time (h), and C is initial concentration of GSH in the donor compartment (mg/mL).

The Kp values confirm that permeability of GSH through sclera was significantly higher than through cornea (*p* < 0.05).

In addition, it was found that the permeability coefficient was lower when the insert was used (both in cornea and sclera). This could be a consequence of the liberation of GSH from the insert. However, it would be better to use this formulation because an ocular insert increases the contact time between the formulation and the eye, thus ensuring sustained release during treatment [28,29]. There are several possibilities for sterilizing such inserts for in vivo use with ultraviolet light, γ-irradiation, or filter-sterilization, while maintaining their physicochemical properties and therapeutic value [58,59,60], so safe application of these inserts to the eye is possible.

Although other research groups have studied the possibility of using progesterone or lipoic acid to increase the production of GSH in the retina [61,62], our suggestion is to administer GSH directly into the eye because it has been previously demonstrated that an increment of GSH in the eye reduces oxidative stress and delays the appearance of eye-related diseases [15,63,64,65,66]. The results obtained in this study provide interesting results for the development of ocular formulations of GSH as an antioxidant for the treatment of eye diseases.

## 4. Conclusions

The results from this study have revealed that the GSH solution is not an irritant to ocular mucosa and is stable in solution (pH 6.5) preserved at 4 °C in darkness for up to one month and the incorporation of Vit C into this solution does not lengthen its preservation. GSH permeated through cornea and sclera, obtaining better results when administered through sclera.

A semisolid ocular insert of GSH was formulated and its evaluation supports the possibility of developing an ocular controlled delivery system of GSH as an antioxidant treatment for ocular diseases in preference to GSH solutions applied as eye drops. As stated earlier, ocular inserts may provide accurate dosing, reduce administration frequency while diminishing visual and systemic side effects, and increasing compliance [28,29].

In the near future, a new generation of ocular delivery systems for antioxidants may reach the market which could represent a significant advance in the therapy for ocular diseases.

There are some data on the pharmacokinetics of GSH. For example, the GSH half-life determined in vivo in *Saccharomyces cerevisiae* was found to be around 90 min [67]. Another study on rats evaluated the pharmacokinetics and distribution of glutathione pegylated liposomes after intravenous and intraperitoneal administration. The area under the curve (AUC) 24 h after administration for liposomal was 983 ± 97 µg h/mL and 665 ± 118 µg h/mL, respectively [56]. In humans, pharmacokinetic parameters were evaluated after 50 mg/kg GSH administration by infusion to adults. In this case, the AUC at 240 min was 1242.8 μM min and the GSH half-life was 10.9 min [68].

Determination of pharmacokinetic parameters in ocular tissues is a major challenge due to the complex anatomy and dynamic physiological barriers of the eye [52,69,70,71,72]. In drug development, human pharmacokinetic parameters are generally assessed after intravenous administration followed by plasma sampling at different time intervals. When drugs are administered to or through the eye and they have a local therapeutic effect, sampling is not feasible. Exceptions could be monitoring drug levels from biopsies, or collection and analysis of aqueous humor from patients undergoing ophthalmic surgical procedures [71].

Taking into the account the complexity of the eye and the degradation of GHS at ocular level, in vivo ocular pharmacokinetic studies will need to be conducted to determine the fate of GSH when administered to the eye using the developed ocular formulation. The results obtained in this study provide interesting results for the development of ocular formulations of GSH as an antioxidant for the treatment of eye diseases.

## Figures and Tables

**Table 1:** GSH ocular semisolid insert composition.

Component	Amount
Polyvinylpyrrolidone K30	600 mg
Hydroxypropyl methylcellulose 4500	400 mg
Polyethylene glycol	0.5 mL
Water	qs for a final volume of 10 mL
Glycerol	25 mg
Glutathione	100 mg

**Table 2:** Stability results obtained for GSH solution and GSH + Vit C solution. Concentration mean as a percentage of original concentration ± SD (*n* = 3).

Solutions	Day	25 °C Light	25 °C Darkness	4 °C Darkness
GSH	2	97.56 ± 3.27	97.30 ± 3.95	99.44 ± 2.35
	7	88.88 ± 1.21	89.37 ± 2.26	99.19 ± 7.78
	14	88.87 ± 1.21	87.44 ± 0.45	98.78 ± 7.29
	30	71.14 ± 0.35	72.92 ± 2.71	92.27 ± 5.78
GSH + Vit C	2	86.81 ± 5.09	95.86 ± 2.54	96.93 ± 8.28
	7	77.17 ± 10.16	86.99 ± 0.39	94.72 ± 6.25
	14	73.87 ± 4.81	78.40 ± 3.00	91.47 ± 4.57
	30	58.71 ± 0.25	64.64 ± 3.20	89.97 ± 1.21

GSH: glutathione. Vit C: vitamin C.

**Table 3:** Permeability coefficient of GSH through cornea and sclera (Kp × 10^3^, cm/h) using different formulations (Mean ± SD; *n* ≥ 3) * *p* < 0.05.

Conditions		Kp × 10^3^ (cm/h)	
		Cornea	Sclera
Solutions	5 mg/mL	6.08 ± 0.83	17.63 ± 3.62 *
	10 mg/mL	6.54 ± 1.79	20.74 ± 2.08 *
Insert	10 mg/mL	3.44 ± 0.64	8.76 ± 1.58
